# Differences in service utilization and barriers among Blacks, Hispanics, and Whites with drug use disorders

**DOI:** 10.1186/1747-597X-4-3

**Published:** 2009-03-13

**Authors:** Brian E Perron, Orion P Mowbray, Joseph E Glass, Jorge Delva, Michael G Vaughn, Mathew Owen Howard

**Affiliations:** 1 University of Michigan, School of Social Work, 1080 S. University Avenue, Ann Arbor, MI 48109, USA; 2 VA Ann Arbor Healthcare System, Serious Mental Illness Treatment Research and Evaluation Center, 11H 2215 Fuller Rd, Ann Arbor, MI 48105, USA; 3 St Louis University, School of Social Work, 3550 Lindell Boulevard, St Louis, MO 63130, USA; 4 University of North Carolina, School of Social Work, Tate-Turner-Kuralt Building, 325 Pittsboro St, Chapel Hill, NC 27599, USA

## Abstract

**Background:**

Treatment for drug use disorders (DUD) can be effective, but only a small proportion of people with DUD seek or receive treatment. Research on racial and ethnic treatment differences and disparities remains unclear. Understanding racial and ethnic differences and disparities in drug treatment is necessary in order to develop a more effective referral system and to improve the accessibility of treatment. The purpose of the current study was to explore the role of race and ethnicity in service utilization.

**Methods:**

Using data from the National Epidemiologic Survey on Alcohol and Related Conditions (NESARC), this study examined racial and ethnic differences in use of 14 types of treatment services for DUD and 27 different treatment barriers among persons who met lifetime criteria for a DUD. Multivariate logistic regression analyses were used to examine service utilization and barriers among the racial and ethnic groups, while adjusting for other sociodemographic and clinical variables.

**Results and discussion:**

Among Blacks, Hispanics and Whites in the overall NESARC sample, approximately 10.5% met criteria for at least one lifetime drug use disorder. Approximately 16.2% of persons with a lifetime DUD received at least one type of service. Overall, this study indicated that Whites were less likely to report receiving help for drug-related problems than Blacks, Blacks used a greater number of different types of services, and no racial and ethnic differences were observed with respect to perceived barriers to drug treatment. However, by examining types of services separately, a complex picture of racial and ethnic differences emerges. Most notably, Whites were most likely to use professional services, whereas Blacks were most likely to use 12-step and clergy. The service use pattern of Hispanics most resembled that of Whites.

**Conclusion:**

While structural barriers to accessing treatment were observed, broad-based educational programs and interventions that are appropriately targeted to racial and ethnic groups remains an important area for prevention and treatment.

## Background

Treatment for drug use disorders (DUD) can be effective (Carr, 2008; Cunningham, 2005), but only a small proportion of people with DUD seek or receive treatment [1,2]. Research on racial and ethnic treatment differences and disparities remains unclear. While Blacks and Hispanics have similar rates of substance use disorders compared to non-Hispanic Whites [3,4], population-based studies show they are less likely to use specialty treatment services [3,5-7]. Other data from publicly-funded programs reveal that Blacks have higher rates of admissions, which can be attributed, in part, to referrals from the criminal justice system [8-10].

Understanding racial and ethnic differences and disparities in drug treatment is necessary in order to develop a more effective referral system and to improve the accessibility of treatments [11]. Racial and ethnic minorities appear to have significantly higher rates of unmet needs for substance use disorders [4] and are less likely to seek or complete treatment [12]. Studying Black and Hispanic populations is particularly important given their anticipated growth and that they make up the majority of the nation's urban population [13]. Considering that HIV/AIDS disproportionately affect Blacks [14] and HIV/AIDS is often associated with and complicated by drug use [15], it is critical to understand the treatment needs of Blacks with DUDs. Studies also consistently find that minority populations experience more adverse health and social consequences related to substances misuse and therefore have more treatment needs [16,17]. This finding may be due to racial or ethnic discrimination, or to issues relating to acculturation stress and community responses to substance use behaviors. Significant increases in substance use disorder treatment gaps between Hispanics and non-Hispanic Whites over the period between 1993 and 2003 were observed [18], a development warranting further monitoring and investigation of services for this population.

Several factors have been examined to help understand different patterns of service utilization among racial and ethnic minorities. Such factors include differential levels of service need [19], help-seeing behaviors [20], co-occurrence of mental illness [21], socioeconomic status [22], and availability of culturally responsive services [23]. Alegria and colleagues [3] found that even when socioeconomic status and culture were taken into account, Blacks reported significantly lower levels of substance use disorder service usage than Whites. Recent service utilization research has also been advanced by focusing on barriers to treatment. Family privacy, lack of knowledge regarding treatment, concerns about stigma, and concerns about medication have been reported as substantial barriers to accessing treatment [24,25]. Understanding how service utilization and barriers vary across different at-risk populations is necessary to develop targeted intervention strategies. To date, however, little information is available regarding the extent to which these barriers vary by race or ethnicity.

Current knowledge of racial or ethnic differences and disparities is limited, as prior research has typically combined mental health and substance abuse services, which masks differences in DUD service needs and factors associated with DUD service utilization [26]. A growing body of research on utilization of services for alcohol use disorders exists [17,27,28], but research on treatment utilization and barriers for DUD remains under-developed. Research focusing on racial and ethnic differences and disparities is necessary to improve service access and utilization for at-risk populations, and to help ensure effective use of limited resources [29]. Increased DUD service utilization is a public health priority as these services can potentially reduce the risk of HIV/AIDS and hepatitis [30], as well as involvement with the correctional system.

The purpose of the current study was to explore the role of race and ethnicity in service utilization. Specifically, this study examined racial and ethnic differences in service utilization rates across 14 types of treatment services for DUD and 27 perceived barriers to service utilization. This study builds on existing research by examining co-morbidity of mental illness and drug use disorder while using nationally representative data.

## Methods

### Subjects, Sampling, and Interviews

This study used data from the National Epidemiologic Survey on Alcohol and Related Conditions (NESARC), which is a nationally representative survey of 43,093 non-institutionalized U.S. residents aged 18 years and older [31]. The NESARC was based on a multistage sampling design, oversampling young adults, Hispanics, and Blacks to obtain reliable statistical estimation in these subpopulations, and to ensure appropriate representation of racial/ethnic groups. The overall response rate was 81%. Data were weighted at the individual and household levels and to adjust for oversampling and non-response on select demographic variables. Data were also adjusted to be representative of the U.S. population as assessed during the 2000 census.

In the administration of this survey, U.S. Census Bureau workers, trained by National Institute on Alcohol Abuse and Alcoholism (NIAAA) staff, administered the Alcohol Use Disorders and Associated Disabilities Interview Schedule – DSM-IV version (AUDADIS-IV). AUDADIS-IV is a structured interview designed for administration by trained lay interviewers. AUDADIS-IV assesses 10 DSM-IV substance use disorders and has evidenced good-to-excellent reliability for the assessment of substance use disorders [32,33]. Descriptions of the NESARC survey, sampling protocol, and related publications are described in detail in prior studies [31-33]

### Measurement

#### Drug use disorders

Participants included those respondents who met DSM-IV lifetime criteria for any non-nicotine drug use disorder (DUD), including abuse or dependence. The specific substances assessed were marijuana, cocaine or crack, tranquilizers, stimulants, painkillers, other prescription drugs, heroin, inhalants or solvents, hallucinogens, and sedatives.

#### Drug treatment utilization

Participants were asked to reply yes or no to the questions: "Have you ever gone anywhere or seen anyone for a reason that was related in any way to your use of medicines or drugs – a physician, counselor, Narcotics Anonymous, or any other community agency or professional?" Did you ever in your life talk to a medical doctor or other professional about your use of drugs?" Participants who endorsed this question were then asked whether they used any of 14 different treatment services. The current study focused on lifetime use of these services.

#### Drug treatment barriers

Participants were asked: "Was there ever a time when you thought you should see a doctor, counselor, or other health professional or seek any other help for your drug use, but you didn't go?" Participants who endorsed this question were then asked about 27 possible barriers to getting help.

#### Sociodemographic variables

Several sociodemographic and clinical variables were assessed in this study: racial/ethnic groups including Whites (non-Hispanic), Blacks, and Hispanics, gender (male, female), living area (urban, rural), marital status (married, separated, never married), personal income (in dollars), age (in years), and employment status (employed, unemployed). Insurance status referred to currently private or public insurance (e.g., Medicare, Medicaid, CHAMPUS, CHAMPVA, VA or other military healthcare). It should be noted that data regarding insurance status at time of diagnosis or when treatment was sought is not available in the NESARC data set.

#### Clinical variables

Five clinical variables were included in this study: lifetime history of a DSM-IV alcohol use disorder (i.e., abuse or dependence), lifetime history of an anxiety disorder (i.e., social phobia, panic disorder with or without agoraphobia, and generalized anxiety disorder), and lifetime history of major depression, personality disorder (i.e., antisocial, avoidant, dependent, obsessive-compulsive, paranoid, schizoid, and histrionic), and polydrug use disorder (i.e., having a lifetime history of more than three non-nicotine or non-caffeine DSM-IV DUD). Note that this definition of polydrug use disorder differs from that of polysubstance-related disorder as defined in DSM-IV (p. 293). Last, it is necessary to note that when assessed for anxiety disorder related symptoms, participants were not assessed for PTSD related symptoms.

### Analytic plan

Analyses were computed using SUDAAN Version 9.0 [34]. This system implements a Taylor series linearization to adjust standard errors of estimates for complex survey sampling design effects including clustered data. Chi-square tests were used to make bivariate comparisons with the study variables. Multivariate logistic regression analyses were used to examine service utilization and barriers among the racial and ethnic groups, while adjusting for other sociodemographic and clinical variables.

## Results

### Overall Sample Characteristics

Among Blacks, Hispanics and Whites in the overall NESARC sample, approximately 10.5% met criteria for at least one lifetime drug use disorder. Whites exhibited the highest rate (11.3%, SE = .32), followed by Blacks (8.7%, SE = .45), and Hispanics (7.2%, SE = .60). These unadjusted results were statistically significant (χ^2 ^[2] = 40.39, p < .001). These persons constitute the current study sample and are described in Table 1. Nearly half of the overall sample was currently unmarried, only a small percentage was 55 years of age or older, nearly 43% earned less than $35,000 annually, approximately 80% met lifetime alcohol use disorder criteria, more than one-third evidenced lifetime anxiety, personality, major depressive and polydrug use disorders and nearly one-in-five was currently unemployed. Regarding subsample characteristics, Blacks were more likely to live in urban areas, have lower incomes, and be unemployed compared to Whites and Hispanics and had the lowest rates of polydrug use disorder. Hispanics tended to be younger than Blacks and Whites. Whites had highest rates of lifetime alcohol and anxiety disorders. Comparatively small differences in rates of major depression and personality disorder were observed across the groups. Few individuals with DUD reported having either private or public medical insurance; therefore, this variable was not included in the multivariate analyses.

**Table 1:** Sociodemographic and clinical characteristics of adults with a lifetime DSM-IV drug use disorder by racial/ethnic groups

Variable	Overall N = 3,887	A. White N = 2,682	B. Black N = 610	C. Hispanic N = 595	χ^2^(p^†^)
Living area		a, b	^a, c^	^b, c^	
Urban	30.68 (1.84)	26.30 (1.75)^ab^	55.71 (3.60)	43.72 (4.11)	**34.10 (< .001)**
Rural	69.32 (1.84)	73.70 (1.75)	44.29 (3.60)	56.28 (4.11)	
					
Marital Status		^a, b^	^a, c^	^b, c^	
Married	54.96 (0.94)	57.66 (1.08)	38.76 (2.34)	47.79 (3.00)	**36.17 (< .001)**
Separated/Divorced	16.65 (0.72)	16.53 (0.81)	20.22 (1.81)	13.70 (1.94)	
Never married	28.39 (0.86)	25.81 (0.97)	41.03 (2.43)	38.51 (2.82)	
					
Personal income**		^a, b^	^a^	^b^	
$0 to $19,999	22.55 (0.83)	20.38 (0.92)	39.16 (2.92)	24.21 (2.63)	**40.05 (< .001)**
$20,000 to $34,999	20.12 (0.82)	20.12 (0.91)	17.20 (1.63)	23.45 (2.30)	
$35,000 to $69,999	32.21 (0.91)	32.48 (1.10)	31.39 (2.48)	30.54 (2.51)	
$70,000 and over	25.12 (1.20)	27.02 (1.38)	12.25 (1.60)	21.80 (2.74)	
					
Has insurance	.53 (.14)	.45 (.16)	1.25 (.68)	.39 (.24)	*
					
Age		a	b	a, b	
18 to 34	41.96 (1.01)	40.12 (1.13)	39.60 (2.47)	62.44 (2.57)	**29.82 (< .001)**
35 to 54	53.00 (1.01)	54.77 (1.11)	54.05 (2.39)	34.71 (2.52)	
55 and over	5.04 (0.40)	5.11 (0.49)	6.35 (0.98)	2.85 (0.70)	
					
Employment status		a, b	a	b	
Employed	82.18 (0.76)	83.68 (0.81)	72.58 (2.62)	78.96 (2.17)	**16.21 (< .001)**
Unemployed	17.82 (0.76)	16.32 (0.81)	27.42 (2.62)	21.04 (2.17)	
					
Lifetime alcohol use disorder		a, b	a, c	b, c	**19.59 (< .001)**
	79.91 (0.83)	81.59 (0.89)	70.63 (2.33)	74.53 (2.45)	
					
Lifetime anxiety disorder		a, b	a, c	b, c	**13.86 (< .001)**
	31.95 (0.97)	33.34 (1.09)	24.00 (1.88)	27.83 (3.11)	
					
Lifetime personality disorder	34.20 (1.00)	33.05 (1.17)	37.30 (2.39)	41.68 (3.11)	6.06 (.055)
					
Major depressive disorder		a, b	a	b	
	36.69 (.097)	37.72 (1.11)	29.32 (2.36)	35.38 (2.63)	**8.75 (.016)**
					
≥ 2s drug use disorders		a, b	a	b	
	35.28 (0.97)	36.17 (1.09)	24.96 (2.07)	38.67 (2.25)	**20.76 (< .001)**

*All Ns in column headings are expressed as unweighted values. All table values are weighted column percentages (standard errors). Values in bold are statistically significant (p < .05). Cells with the same letters indicate group differences based on pair-wise post-hoc comparisonsusing 2 × 2 chi-square tests (p < .05). *Differences not tested due to low cell count. **Measured in dollars per year.

### Differences in Types of Services Used

Of the sample of persons with a lifetime drug use disorder, approximately 16.2% (SE = .75) had received at least one type of service. Blacks were most likely to receive services (20.8%, SE = 2.05), followed by Hispanics (17.3%, SE = 2.25) and Whites (15.5%, SE = .81). Whites had significantly lower rates of treatment compared to Blacks and Hispanics, but rates between Blacks and Hispanics did not differ significantly.

Table 2 provides a summary of treatment utilization for DUD by types of service. Overall, the most commonly utilized types of services used were 12-step programs (62.6%), private physician or professionals (55.1%), and drug/alcohol rehabilitation programs (51.5%). Chi-square tests were used to compare differences in rates across each racial and ethnic group. Significant overall group differences were observed for 4 of the 14 service types, including use of 12-step meetings, private professionals, drug/alcohol rehabilitation programs, and outpatient clinics. To better understand where specific racial and ethnic differences existed, pair-wise comparisons were conducted where overall group differences were observed. These analyses indicated that Blacks had significantly higher rates of use of 12-step meetings, drug/alcohol rehabilitation programs, outpatient clinics, inpatient wards, clergy services, and other types of services compared to Whites. Whites, on the other hand, exhibited significantly higher rates of professional service use. Compared to Whites, Hispanics used inpatient wards, clergy services, and other types of services at higher rates, and used private professionals and outpatient clinics at lower rates.

**Table 2:** Types of services used by adults with a DSM-IV lifetime drug use disorder who used at least one type of service

		Race/ethnicity comparisons			
		
Treatment type	Overall N = 600 % (SE)	White N = 417 % (SE)	Black N = 135 % (SE)	Hispanic N = 108 % (SE)	χ^2 ^(p^†^)
Narcotics/cocaine/alcoholics Anonymous or any 12 step meeting	62.64 (2.62)	60.86 (3.08)^a^	79.67 (4.36)^a^	54.19 (6.98)	**11.34 (.005)**
					
Private physician, psychiatrist, psychologist, social worker, or other professional	55.07 (2.45)	57.63 (2.95)^a, b^	39.45 (4.93)^a^	54.86 (6.19)^b^	**7.94 (.024)**
					
Drug/alcohol rehabilitation program	51.45 (2.67)	49.93 (3.07)^a^	65.85 (4.45)^a^	44.59 (7.55)	**8.45 (.019)**
					
Drug/alcohol detoxification ward/clinic	39.18 (2.74)	38.33 (2.98)	48.03 (5.36)	34.37 (7.33)	3.56 (.177)
					
Outpatient clinic, including outreach and day/partial patient program	36.66 (2.47)	34.36 (2.87)^a, b^	54.74 (5.36)^a^	31.35 (5.36)^b^	**10.65 (.007)**
					
Inpatient ward of psychiatric/general hospital or community mental health program	31.02 (2.10)	28.51 (2.41)	39.75 (5.16)	40.26 (7.27)	5.46 (.072)
					
Emergency room because of medicine/drug use	23.35 (1.86)	22.44 (2.14)	22.01 (4.41)	32.90 (8.26)	1.38 (.506)
					
Family services or other social service agency	21.69 (2.13)	20.29 (2.49)	27.07 (5.27)	26.20 (6.26)	2.03 (.368)
					
Clergyman, priest, or rabbi because of medicine/drug use	20.31 (1.89)	18.27 (2.25)	32.77 (4.80)	20.53 (5.54)	6.15 (.053)
					
Halfway house because of medicine/drug use	12.56 (1.71)	11.75 (1.92)	17.15 (4.60)	13.10 (4.05)	1.30 (.526)
					
Other agency or professional	11.75 (1.51)	10.06 (1.81)	20.03 (3.99)	14.68 (3.79)	5.16 (.084)
					
Employment assistance program (EAP)	9.49 (1.47)	9.45 (1.80)	13.95 (3.52)	3.73 (1.97)	6.14 (.053)
					
Crisis center because of medicine/drug use	6.37 (1.46)	5.82 (1.68)	11.07 (3.91)	4.54 (2.02)	2.05 (.364)
					
Methadone maintenance program	5.28 (1.10)	4.40 (1.20)	8.92 (2.65)	7.74 (3.46)	3.47 (.185)

Note: All Ns in column headings are expressed as unweighted values. All table values are weighted column percentages (standard errors). Values in bold are statistically significant (p < .05). ^†^Tests of differences based on chi-square. Cells with the same letters indicate group differences based on pair-wise post-hoc comparison using 2 × 2 chi-square tests (p < .05).

A series of multivariate logistic regression models were used to further examine the significant associations identified in Table 2. Each service type was used as an outcome variable. Race and ethnicity were entered into the model as the primary independent variable, along with other potentially confounding sociodemographic (e.g., gender, rural vs. urban residence, marital status, personal income, age) and clinical characteristics (e.g., lifetime alcohol use disorder, polydrug use disorder, anxiety disorder, personality disorder, and major depressive disorder). For analysis pertaining to each type of service received, the reference group for race/ethnicity was changed to facilitate intergroup comparisons.

Adjusted odds ratios (OR) and 95% confidence intervals (CI) for race/ethnicity are provided in Table 3. For purposes of brevity, values for the control variables are not reported. Many significant race and ethnic associations with service utilization were observed in the multivariate analysis. The differential trends comparing Blacks and Whites were consistent with the bivariate analyses. Blacks were almost three times more likely to use 12-step programs compared to Whites (OR = 2.96, 95% CI = 1.53 – 5.72). Also, Blacks were between two and three times more likely to use drug/alcohol rehabilitation programs, inpatient wards, clergy services, and other types of services compared to Whites. However, Whites were also almost twice as likely to use private professionals compared to Blacks (OR = 1.79, 95% CI = 1.08 – 2.96). No significant differences were observed comparing Hispanics and Whites. Compared to Hispanics, Blacks were significantly more likely to use 12-step meetings (OR = 3.31, 95% CI = 1.50 – 7.31), rehabilitation programs (OR = 2.83, 95% CI = 1.42 – 5.65), and outpatient clinics (OR = 2.83, 95% CI = 1.42 – 5.65).

**Table 3:** Multivariate associations between race/ethnicity and service utilization among adults with a lifetime drug use disorder who used at least one type of service (N = 600)

Treatment type	Blacks Hispanics (Whites as reference) AOR (95% CI)	Whites Hispanics (Blacks as reference) AOR (95% CI)	Whites Blacks (Hispanics as reference) AOR (95% CI)	Adjusted Wald F (p)
Narcotics/cocaine/alcoholics Anonymous or any 12 step meeting	**2.96 (1.53–5.72)** .45 (0.18–1.15)	**.34 (.17 – .65)** .30 (.14 – .67)	1.21 (.62 – 2.03) **3.31 (1.50 – 7.31)**	6.07 (.004)
				
Private physician, psychiatrist, psychologist, social worker, or other professional	**.56 (.34 – .93)** .85 (.49 – 1.48)	**1.79 (1.08 – 2.96)** 1.52 (.77 – 2.99)	1.18 (.68 – 2.04) .66 (.33 – 1.29)	2.74 (.072)
				
Drug/alcohol rehabilitation program	**2.19 (1.34 – 3.57)** .77 (.42 – 1.44)	**.46 (.28 – .74)** **.35 (.18 – .71)**	1.29 (.70 – 2.39) **2.83 (1.42 – 5.65)**	6.79 (.002)
Outpatient clinic, including outreach and day/partial patient program	**2.23 (1.26 – 3.94)** .76 (.41 – 1.39)	.**45 (.25 – .79)** **.34 (.17 – .70)**	1.32 (.72 – 2.41) **2.94 (1.44 – 6.00)**	5.31 (.007)
				
Inpatient ward of psychiatric/general hospital or community mental health program	**.56 (.34 – .93)** .85 (.49 – 1.48)	**1.79 (1.08 – 2.96)** 1.52 (.77 – 2.99)	1.18 (.68 – 2.04) .66 (.33 – 1.29)	4.61 (.013)
				
Clergyman, priest, or rabbi because of medicine/drug use	**2.79 (1.52 – 5.14)** 1.39 (.65 – 3.01)	**.36 (.19 – .66)** .50 (.21 – 1.19)	.72 (.33 – 1.55) 2.00 (.84 – 4.77)	5.55 (.006)
Other agency or professional	**2.33 (1.13 – 4.77)** 1.49 (.71 – 3.16)	**.43 (.21 – .88)** .64 (.27 – 1.55)	.67 (.32 – 1.41) 1.56 (.64 – 3.75)	2.70 (.075)
Employment assistance program (EAP)	1.68 (.72 – 3.91) .57 (.17 – 1.93)	.59 (.26 – 1.38) .34 (.09 – 1.25)	1.76 (.52 – 6.00) 2.97 (.80 – 11.01)	1.56 (.217)

Note: N = 3,887. AOR = Adjusted odds ratio. CI = confidence intervals. All models were adjusted for living area (urban vs. rural), marital status, annual personal income, age, employment status, lifetime alcohol use disorder, lifetime anxiety disorder, lifetime personality disorder, lifetime major depression and lifetime polydrug use disorder. All values in bold are statistically significant based on an confidence interval that does not include the value 1.0.

### 3.3 Differences in Number of Different Services Used

Among adults with a lifetime DUD who used services, the overall number of different types of services used ranged from one to eight (Mean = 2.8, SE = .09). Blacks used a larger number of different types of services (Mean = 3.5, SE = .23) compared to Whites (2.7, SE = .10) and Hispanics (2.6, SE = .21) (F = 4.98, p = .010). Whites and Hispanics exhibited no differences.

### Differences in Types of Treatment Barriers

Among persons with DUD, approximately 12.4% reported having a need for treatment but did not receive any services. Persons who felt they needed but did not receive treatment were queried about 27 different types of possible barriers encountered.

Table 4 summarizes rates of each barrier encountered among adults with a lifetime drug use disorder. The most frequently cited barriers included thinking that the problem should be handled alone (41.7%), thinking the problem would get better by itself (37.2%), too embarrassed to discuss the problem (25.8%), and couldn't afford to pay the bill (20.8%). Chi-square analyses did not reveal any significant racial or ethnic group difference.

**Table 4:** Perceived barriers to treatment among adults with a lifetime DSM-IV drug use disorder and perceived unmet need for treatment

Drug Use Treatment Barrier	Overall (N = 511) % (SE)	White (N = 310) % (SE)	Black (N = 107) % (SE)	Hispanic (N = 94) % (SE)	χ^2 ^(p)
Thought should be strong enough to handle alone	41.65 (2.69)	42.09 (3.25)	43.12 (5.76)	36.44 (7.63)	.47 (.793)
					
Thought the problem would get better by itself	37.21 (2.82)	36.56 (3.43)	40.90 (5.79)	36.96 (7.91)	.44 (.804)
					
Was too embarrassed to discuss it with anyone	25.80 (2.38)	24.79 (2.85)	32.66 (5.39)	23.81 (6.70)	1.65 (.444)
					
Couldn't afford to pay the bill	20.77 (2.31)	21.08 (2.68)	20.13 (5.57)	19.34 (5.67)	.08 (.963)
Didn't want to go	20.72 (2.07)	21.81 (2.58)	17.74 (4.53)	16.78 (5.48)	.99 (.612)
					
Stopped using a drug or medicine on my own	18.46 (1.87)	19.49 (2.25)	15.63 (4.13)	14.79 (5.11)	1.21 (.549)
					
Afraid of what boss, family, friends or others might think	18.35 (2.36)	18.88 (2.94)	14.10 (4.35)	20.26 (6.18)	1.02 (.602)
					
Hated answering personal questions	17.35 (1.90)	16.20 (2.35)	22.94 (5.12)	18.18 (5.79)	1.42 (.495)
Didn't think anything could help	16.71 (1.98)	17.56 (2.56)	13.94 (3.70)	14.25 (4.15)	.96 (.620)
Didn't think medicine or drug problem was serious enough	16.47 (2.11)	17.04 (2.55)	12.74 (3.74)	17.44 (5.02)	.95 (.624)
					
Wanted to keep using medicine or drug	15.99 (1.83)	16.29 (2.32)	16.89 (4.51)	12.50 (3.84)	.72 (.698)
					
Afraid they would put me into the hospital	13.77 (1.95)	13.94 (2.33)	13.86 (3.90)	12.36 (4.64)	.10 (.953)
					
Wanted to go, but health insurance didn't cover	10.46 (1.61)	9.74 (1.91)	9.42 (3.57)	17.21 (5.25)	1.80 (.412)
					
Didn't have the time	9.92 (1.43)	8.29 (1.71)	12.62 (4.53)	18.21 (6.15)	3.10 (.220)
					
Didn't know any place to go for help	8.34 (1.45)	6.83 (1.63)	13.29 (3.98)	12.70 (4.70)	3.43 (.188)
Afraid of the treatment they would give me	8.21 (1.30)	6.49 (1.47)	12.21 (4.13)	15.37 (5.75)	3.15 (.215)
Family thought I should go, but didn't think it was necessary	7.00 (1.54)	6.72 (1.66)	4.68 (2.24)	12.20 (5.66)	1.5 (.478)
					
Tried getting help before and it didn't work	5.63 (1.09)	4.64 (1.18)	9.73 (3.35)	7.34 (3.80)	.09 (.956)
Afraid I would lose my job	4.83 (1.10)^†^	4.72 (1.27)	4.68 (2.04)	5.85 (3.57)	-
Other reason	4.66 (1.36)	3.68 (1.02)	3.53 (1.29)	13.44 (10.06)	.82 (.664)
					
Didn't have any way to get there	4.60 (1.10)	4.43 (1.31)	4.08 (1.98)	6.53 (3.99)	.30 (.850)
Friends or family helped me stop using a medicine or drug	4.27 (.89)^†^	4.47 (1.09)	1.69 (1.03)	6.34 (3.67)	-
					
Hours were inconvenient	3.80 (1.17)^†^	3.69 (1.37)	4.11 (3.08)	4.20 (3.20)	-
Had to wait too long to get into program	3.22 (.91)^†^	3.36 (1.14)	4.42 (1.99)	.52 (.53)	**-**
					
Couldn't arrange for child care	1.32 (.48)^†^	1.43 (.59)	1.12 (.84)	.74 (.75)	-
A member of my family objected	.52 (.35)^†^	.11 (.11)	.00 (.00)	4.31 (3.25)	-
Can't speak English very well	.34 (.27)^†^	.11 (.11)	.00 (.00)	2.50 (2.49)	-

Note: ^†^Chi-square tests not performed due to cell counts < 5.

### Differences in Number of Treatment Barriers

The number of barriers reported ranged from 1 to 26, with an average of 3.4 (Whites, Mean = 3.3; Blacks, Mean = 3.5, Hispanic, Mean = 3.7). No significant differences in the number of barriers encountered were observed across the racial/ethnic groups (F = .16, p = .854).

## Discussion

This study examined racial and ethnic differences in the utilization of drug treatment services and barriers to receiving treatment. Notable strengths of this study included using data from a nationally representative survey that is among the largest and most current psychiatric epidemiologic studies. Using this same data source, other studies have examined factors associated with substance use disorder treatment utilization [35,36]. However, to date, no studies have provided a comprehensive examination of differences across three major racial and ethnic groups, with a focus on differences in specific types of treatment services used and barriers to treatment.

Overall, this study indicated that Whites were less likely to report receiving help for drug-related problems than Blacks, Blacks used a greater number of different types of services, and no racial and ethnic differences were observed with respect to perceived barriers to drug treatment. However, by examining types of services separately, a complex picture of racial and ethnic differences emerges. Provided below is a discussion of the major findings vis-à-vis the current study methodology and the broader literature on treatment for substance-related problems.

### Service utilization

Approximately one-in-six persons with a lifetime drug use disorder reported receiving some type of help for drug-related problems. Whites had lower rates of treatment compared to Blacks and Latinos. Although this finding contrasts with prior services research [3,5,6], it is important to consider the between group variation as it relates to type of treatment received. For example, Blacks were significantly less likely to rely on professional services compared to Whites, but were significantly more likely to receive non-professional services, including 12-step meetings and church-related support. This finding underscores the importance of how services are measured, given the diversity in types of treatment and help that are available. Rates of drug/alcohol rehabilitation program utilization were also higher for Blacks compared to Whites. This suggests that Blacks may lack access to preventive resources to reduce the consequences of drug use problems, as suggested by prior researchers [23]. Moreover, using the same data source as the current study, Keyes et al. [35] found Blacks to have higher levels of drug use disorder symptoms compared to Whites, showing a higher level of potential service need. Potentially, differences in the way that services are utilized may be based on geographic factors. For example, a recent study by Velez and colleagues [37] found that cities with higher proportions of African Americans and Latinos have greater access to specialty services, such as services for people who are older, gay or lesbian, or who have HIV. While their study did not analyze 12-step programs, it is possible that a similar trend exists, where 12-step programs are more prevalent in cities with a higher racial/ethnic diversity.

Latinos exhibited few significant differences in drug treatment utilization compared to Whites and Blacks. Although the sample size of Latinos is only slightly smaller than Blacks, it is important to note that the standard errors are consistently larger for this group, which is due to a smaller sample size and possibly significant within group differences. Future research will consider additional subgroup analyses, focusing subgroups of disorders (e.g., separating disorders of abuse from dependence) and other sociodemographic factors (e.g., income levels). Considering that Blacks overwhelmingly use 12-step programs as a source of treatment, this highlights issues of the potential lack of cultural relevance, availability, or accessibility of other types of services. At the same time, 12-step meetings and professional services were the most commonly used services among Latinos for drug use disorders, which was the pattern for Whites.

Overall, these differences in patterns of utilization show that all three racial and ethnic groups use 12-step meetings at a high rate, but these services are typically in conjunction with other types of help. Identifying differences across racial and ethnic groups also guides our understanding regarding treatment preferences and accessibility. Although the current data do not provide an opportunity to examine these concepts, the role of barriers to treatment described in this study does offer initial guidance.

### Treatment Barriers

Overall, racial and ethnic groups showed no differences with respect to numbers or different types of barriers to treatment encountered. One possibility is that there are minimal differences with respect to internal and external barriers to accessing drug treatment services. This is evidenced by the small differences in rates across the racial and ethnic groups. The most common barriers to treatment across the groups were internal barriers, including thinking one should be strong enough to handle the problem alone, thinking the problem would get better by itself, and being too embarrassed to discuss it with anyone. Thus, even if there are structural barriers preventing persons from accessing drug treatment, these data underscore the importance of greater public awareness across all ethnic groups. This includes increased knowledge regarding the course of drug use disorders and education that will address stigma.

Although statistically significant differences were not observed, a few important trends in the data still need to be considered. For several barriers, Whites experienced rates that were approximately half those of Blacks and Latinos, including not having health insurance to cover expenses, not having the time, not knowing where to go for help, and fear of treatment. Furthermore, individuals with a perceived need for treatment but who have not used treatment are difficult to capture in general community samples, which increases the difficulty of studying treatment barriers from a population perspective. The problem of identifying significant differences across racial and ethnic groups is further compounded by the need for large samples. Therefore, it is important to consider issues of statistical power and, at the same time, be cautious of dismissing potentially important trends.

### Limitations and Future Directions

It is important to consider these findings in the context of the study limitations. Foremost, regional variation is an important issue that we were not able to take into consideration in this paper. More specifically, [Perron BE et al, Mapping availability of outpatient substance abuse treatment programs in urban areas, submitted] used geographic information systems to show significant regional differences in the availability of outpatient substance use disorder treatment programs in urban areas. The current study revealed that Blacks were more likely to use outpatient treatment programs. Overall trends in this study do not reveal important regional differences and within group differences. Additionally, legal coercion and other forms of social pressure are common place in treatment for substance use disorders and may differentially affect services use [38]. Coercion as it relates to service utilization was not assessed in the NESARC. Thus, the results of this study need to be considered cautiously.

Another limitation of this study was the lack of data to examine the extent to which respondents felt that services were not beneficial. While Blacks may have utilized multiple service types that have strong community and network connections, it is also plausible that they relied on multiple options due to needing additional help. The extent to which respondents believed these services were helpful was not assessed. However, other data sources, such as the National Survey of American Life and NLAAS, may help answer these questions. Although they are not as large as the NESARC, they do provide the opportunity to examine issues of services and perceptions of helpfulness among comparably large samples of Blacks and Latinos.

Large-scale epidemiologic studies provide the opportunity to identify trends, which can help us hone in on problem areas and make subsequent research initiatives more effective and efficiency. A greater understanding of racial and ethnic differences will require studies of service utilization addressing more region-specific areas, while also using more nuanced measures of services in order to strengthen the empirical picture on racial and ethnic differences. Finally, it is also important to consider the importance of ongoing surveillance of the problem, in light of a service system and funding mechanism that are continually changing.

## Conclusion

This study found no differences in overall rates of drug treatment utilization across racial and ethnic groups. However, notable differences were observed in relation to types of services used. Blacks were more likely to use non-professional services, whereas Whites were more likely to use professional services. Latinos had similar patterns of utilization compared to Whites. No significant racial and ethnic differences were observed with respect to barriers to treatment. Over one-third of respondents who did not use treatment reported thinking they could handle the problem alone or it would get better, and approximately one-fourth were embarrassed to discuss their problem with anyone.

Study results are also suggestive of treatment and policy initiatives. Increasing access to treatment options is an important issue, and strategically locating treatments in underserved communities is necessary but not sufficient. Professional services will have to be made affordable and culturally responsive to better serve Black communities. Such recommendations may be difficult to carry out during periods in which funding is severely limited due to an economic crisis. However, the potential long-term savings are significant when community based treatments reduce drug-related emergency room visits, health problems, and involvement with the criminal justice system. Additionally, while structural barriers to accessing treatment were observed, broad-based educational programs that are appropriately targeted to racial and ethnic groups remain an important area for prevention and treatment. This would help address issues of stigma that were common treatment barriers observed among all racial and ethnic groups.

## Competing interests

The authors declare that they have no competing interests.

## Authors' contributions

The first two authors (BEP and OPM) conceptualized the study and solicited feedback from the other authors. The first two authors conducted the analyses and worked collaboratively with the other members of the research team to interpret the results, and prepare and revise the manuscript.
